# Supplementary material for: Imaging immunomodulatory treatment responses in a multiple sclerosis mouse model using hyperpolarized 13C metabolic MRI
Source: Commun Med (Lond). 2023 May 22;3:71. doi: 10.1038/s43856-023-00300-1 (PMC10202949; doi:10.1038/s43856-023-00300-1)
Supplement: Supplementary file 1 — Description of Additional Supplementary Files [file 43856_2023_300_MOESM1_ESM.pdf]

Description of Additional Supplementary File

File Name: Supplementary Data 1

Description: Data to reproduce Figure 1.

File Name: Supplementary Data 2

Description: Data to reproduce Figure 2.

File Name: Supplementary Data 3

Description: Data to reproduce Figure 3.

File Name: Supplementary Data 4

Description: Data to reproduce Supplementary Figure 1.

File Name: Supplementary Data 5

Description: Data to reproduce Supplementary Figure 2.
