# Supplementary material for: Imaging immunomodulatory treatment responses in a multiple sclerosis mouse model using hyperpolarized 13C metabolic MRI
Source: Commun Med (Lond). 2023 May 22;3:71. doi: 10.1038/s43856-023-00300-1 (PMC10202949; doi:10.1038/s43856-023-00300-1)
Supplement: Supplementary file 2 — Supplementary Information [file 43856_2023_300_MOESM2_ESM.pdf]

Supplementary Information

Imaging immunomodulatory treatment responses in a multiple sclerosis mouse model using hyperpolarized ^{13}C metabolic MRI

Caroline Guglielmetti[1,2*], Christian Cordano[3], Chloé Najac[4], Ari Green[3], Myriam M. Chaumeil[1,2*]

Affiliations:

[1] Department of Physical Therapy and Rehabilitation Science, University of California San Francisco, San Francisco, CA, USA.

[2] Department of Radiology and Biomedical Imaging, University of California San Francisco, San Francisco, CA, USA.

[3] Weill Institute for Neurosciences, Department of Neurology, University of California at San Francisco, San Francisco, CA, USA.

[4] C.J. Gorter MRI Center, Department of Radiology, Leiden University Medical Center, Leiden, The Netherlands.

*Corresponding authors.

E-mail: caroline.guglielmetti@ucsf.edu

E-mail: myriam.chaumeil@ucsf.edu

Supplementary Figure 1. ^{13}C lactate/pyruvate mean z-score detects therapy responses.

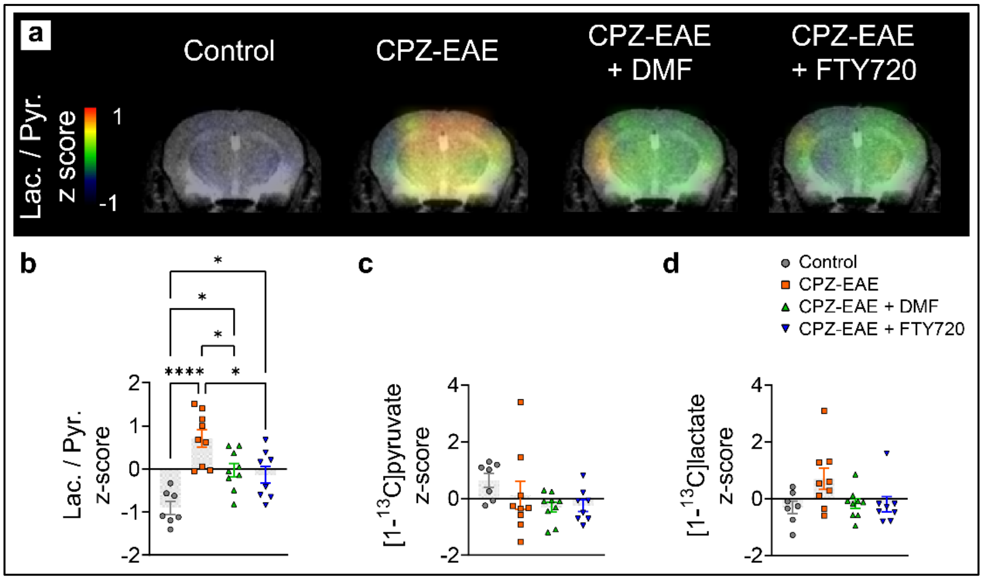

(a) ^{13}C lactate/pyruvate mean z-score color maps overlaid on a representative T_2-weighted image for the control, CPZ-EAE, CPZ-EAE + DMF and CPZ-EAE + FTY720 groups. **(b)** Quantitative analyses revealed significant increase of the ^{13}C lactate/pyruvate z-score in CPZ-EAE mice compared to Control mice. Both DMF and FTY720 showed lower ^{13}C lactate/pyruvate z-score compared to untreated CPZ-EAE mice. No significant differences were observed between groups for **(c)** the hyperpolarized [1-^{13}C]pyruvate and **(d)** [1-^{13}C]lactate z-scores. We noted a trend for an increase hyperpolarized [1-^{13}C]lactate z-score in CPZ-EAE mice compared to control mice (*p = 0.079*). Abbreviations: Cuprizone and experimental autoimmune encephalomyelitis (CPZ-EAE), Dimethyl fumarate (DMF), Fingolimod (FTY720), lactate-to-pyruvate ratio (Lac. / Pyr.). Data is shown as mean ±standard error. Control are indicated by grey circles (n = 7 mice), CPZ-EAE by orange rectangles (n = 9 mice), CPZ-EAE + DMF by green triangles (n = 9 mice), and CPZ-EAE + FTY720 by blue inverted triangles (n = 8 mice). (*$p \leq 0.05$, ****$p \leq 0.0001$). Data to reproduce this figure are included in Supplementary Data 4.

Supplementary Figure 2. Evaluation of myelin basic protein levels following immunomodulatory therapies in the CPZ-EAE model.

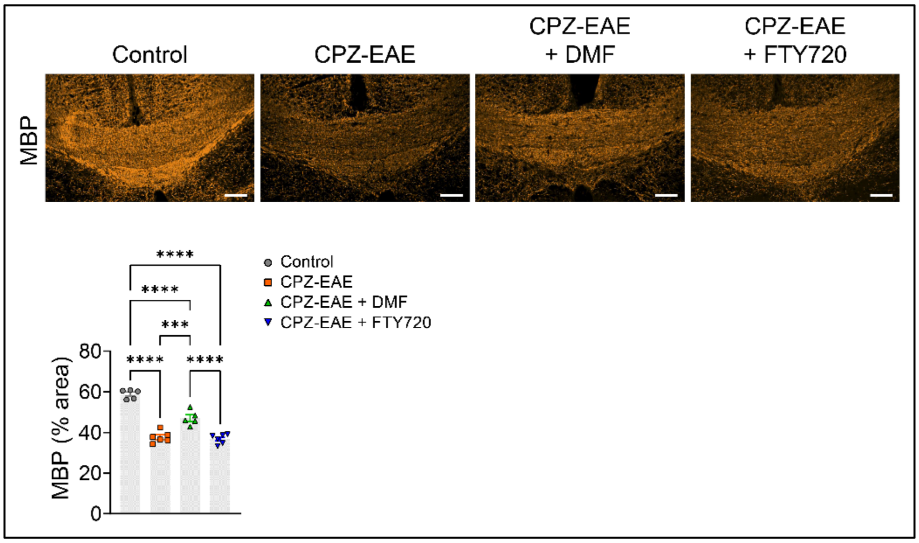

Representative immunofluorescence staining of myelin basic protein (MBP, orange) in the corpus callosum from control (grey circles), CPZ-EAE (orange rectangles), CPZ-EAE + DMF (green triangles), and CPZ-EAE + FT720 (blue inverted triangles) mice**.** Demyelination was confirmed by a 1.6 fold decrease MBP in untreated CPZ-EAE mice compared to control mice ($p < 0.0001$). DMF treated mice displayed an increased MBP content (+1.25 fold, $p = 0.0002$) compared to untreated CPZ-EAE mice, while no change was observed following FTY720. Abbreviations: Cuprizone and experimental autoimmune encephalomyelitis (CPZ-EAE), Dimethyl fumarate (DMF), Fingolimod (FTY720), myelin basic protein (MBP). Data is shown as mean ±standard error. Control are indicated by grey circles ($n = 5$ mice), CPZ-EAE by orange rectangles ($n = 6$ mice), CPZ-EAE + DMF by green triangles ($n = 5$ mice), and CPZ-EAE + FTY720 by blue inverted triangles ($n = 6$ mice). (***$p \leq 0.001$, ****$p \leq 0.0001$). Scale bar is 100 μm. Data to reproduce this figure are included in Supplementary Data 5.
