# Supplementary material for: Imaging immunomodulatory treatment responses in a multiple sclerosis mouse model using hyperpolarized 13C metabolic MRI
Source: Commun Med (Lond). 2023 May 22;3:71. doi: 10.1038/s43856-023-00300-1 (PMC10202949; doi:10.1038/s43856-023-00300-1)
Supplement: Supplementary file 9 — Reporting Summary [file 43856_2023_300_MOESM9_ESM.pdf]

nature portfolio

Corresponding author(s): Caroline Guglielmetti
Myriam M. Chaumeil

Last updated by author(s): Apr 6, 2023

Reporting Summary

Nature Portfolio wishes to improve the reproducibility of the work that we publish. This form provides structure for consistency and transparency in reporting. For further information on Nature Portfolio policies, see our [Editorial Policies](#) and the [Editorial Policy Checklist](#).

Statistics

For all statistical analyses, confirm that the following items are present in the figure legend, table legend, main text, or Methods section.

n/a	Confirmed	
☐	☒	The exact sample size (n) for each experimental group/condition, given as a discrete number and unit of measurement
☐	☒	A statement on whether measurements were taken from distinct samples or whether the same sample was measured repeatedly
☐	☒	The statistical test(s) used AND whether they are one- or two-sided<br>*Only common tests should be described solely by name; describe more complex techniques in the Methods section.*
☒	☐	A description of all covariates tested
☐	☒	A description of any assumptions or corrections, such as tests of normality and adjustment for multiple comparisons
☐	☒	A full description of the statistical parameters including central tendency (e.g. means) or other basic estimates (e.g. regression coefficient) AND variation (e.g. standard deviation) or associated estimates of uncertainty (e.g. confidence intervals)
☐	☒	For null hypothesis testing, the test statistic (e.g. F, t, r) with confidence intervals, effect sizes, degrees of freedom and P value noted<br>*Give P values as exact values whenever suitable.*
☒	☐	For Bayesian analysis, information on the choice of priors and Markov chain Monte Carlo settings
☒	☐	For hierarchical and complex designs, identification of the appropriate level for tests and full reporting of outcomes
☒	☐	Estimates of effect sizes (e.g. Cohen's d, Pearson's r), indicating how they were calculated

Our web collection on statistics for biologists contains articles on many of the points above.

Software and code

Policy information about availability of computer code

Data collection	MRI data were collected using VNMJ software. Enzyme activity assays were collected using Magellan software. Fluorescent widefield images were captured on an inverted Nikon TI microscope run with NIS-Elements 5.20.21 (Nikon) and confocal images were captured on an inverted Nikon Ti microscope run using Micro Manager 2.0 Gamma.
Data analysis	HP 13C MRSI datasets were analyzed using the SIVIC software (http://sourceforge.net/apps/trac/sivic/) and custom-built programs written in MATLAB (MATLAB R2011b, The MathWorks Inc.). Quantitative analyses of immunofluorescence images were performed using NIH ImageJ analysis software (v1.51n). Statistical analyses were performed using GraphPad Prism version 9.1.2.

For manuscripts utilizing custom algorithms or software that are central to the research but not yet described in published literature, software must be made available to editors and reviewers. We strongly encourage code deposition in a community repository (e.g. GitHub). See the Nature Portfolio guidelines for submitting code & software for further information.

Data

Policy information about availability of data

All manuscripts must include a data availability statement. This statement should provide the following information, where applicable:

- Accession codes, unique identifiers, or web links for publicly available datasets
- A description of any restrictions on data availability
- For clinical datasets or third party data, please ensure that the statement adheres to our policy

The datasets generated during and/or analysed during the current study are available from the corresponding author on reasonable request. Custom computer code or algorithm used to generate results that are reported in the paper will be made available from the corresponding author upon reasonable request.The source data needed to reproduce the graphs shown in Figures 1, 2, 3 and Supplementary Figures 1 and 2 can be found in Supplementary Data 1, 2, 3, 4 and 5, respectively.

Human research participants

Policy information about studies involving human research participants and Sex and Gender in Research.

Reporting on sex and gender	NA
Population characteristics	NA
Recruitment	NA
Ethics oversight	NA

Note that full information on the approval of the study protocol must also be provided in the manuscript.

Field-specific reporting

Please select the one below that is the best fit for your research. If you are not sure, read the appropriate sections before making your selection.

☒ Life sciences ☐ Behavioural & social sciences ☐ Ecological, evolutionary & environmental sciences

For a reference copy of the document with all sections, see nature.com/documents/nr-reporting-summary-flat.pdf

Life sciences study design

All studies must disclose on these points even when the disclosure is negative.

Sample size	No sample size calculation were performed. We based our sample size on previously published studies by our group.
Data exclusions	No data were excluded
Replication	No replication were performed
Randomization	The samples were allocated into experimental groups randomly by the performers.
Blinding	Experimenters were not blind, data analysis was not blind. All data analysis steps were performed in the exact same way independently of the experimental group

Reporting for specific materials, systems and methods

We require information from authors about some types of materials, experimental systems and methods used in many studies. Here, indicate whether each material, system or method listed is relevant to your study. If you are not sure if a list item applies to your research, read the appropriate section before selecting a response.

Materials & experimental systems

n/a	Involved in the study
☐	☒ Antibodies
☒	☐ Eukaryotic cell lines
☒	☐ Palaeontology and archaeology
☐	☒ Animals and other organisms
☒	☐ Clinical data
☒	☐ Dual use research of concern

Methods

n/a	Involved in the study
☒	☐ ChIP-seq
☒	☐ Flow cytometry
☐	☒ MRI-based neuroimaging

Antibodies

Antibodies used: a primary chicken anti-myelin basic protein (MBP) antibody (AB9348; 1:200 dilution; Millipore) with a secondary donkey anti-chicken DyLight 549 antibody (703-506-155, 1:1,000 dilution; Jackson); a primary rabbit anti-Iba-1 antibody (019-19741, 1:500 dilution; Wako) with a secondary donkey anti-rabbit Alexa Fluor 555 (A31572, 1:1,000 dilution; Invitrogen); a primary mouse anti-PDK1 antibody (AB110025, 1:100 dilution; Abcam) with a secondary goat anti-mouse Alexa Fluor 488 (A11017, 1:100 dilution; Invitrogen); a primary rabbit anti-GFAP (AB7779, 1:500 dilution; Abcam) with a secondary goat anti-rabbit Alexa Fluor 488 (A11008, 1:1,000 dilution; Invitrogen); a rabbit anti-fibrinogen (A0080, 1:200 dilution; Dako) with donkey anti-rabbit Alexa Fluor 555 (A31572, 1:600 dilution; Invitrogen); a rat anti-CD3 (MCA500G, 1: 400 dilution; BioRad) with goat anti-rat Alexa Fluor 488 (A11006, 1:800 dilution; Invitrogen); a primary rat anti-CD68 (BioRad, MCA1957, 1:100 dilution) with a secondary anti-rat AF488 (Life Technologies, A11006, 1:200).

Validation: Antibodies have been validated by the manufacturer and have been previously published in peer-reviewed papers

Animals and other research organisms

Policy information about studies involving animals; ARRIVE guidelines recommended for reporting animal research, and Sex and Gender in Research

Laboratory animals: Female C57BL/6 mice (The Jackson Laboratory; 000664) were used for this study

Wild animals: NA

Reporting on sex: Multiple sclerosis is a disease that mostly affects women (2/3), therefore female mice have been used in this study

Field-collected samples: NA

Ethics oversight: All animal research was approved by the Institutional Animal Care and Use Committee of the University of California, San Francisco

Note that full information on the approval of the study protocol must also be provided in the manuscript.

Magnetic resonance imaging

Experimental design

Design type: Single time point acquisition of metabolic hyperpolarized 13C MR spectroscopy data together with T2-weighted MRI, single timepoint acquisition of T1 weighted contrast enhanced MRI

Design specifications: 1 acquisition of dataset per session

Behavioral performance measures: NA

Acquisition

Imaging type(s): Hyperpolarized 13C MRS imaging, T2-weighted MRI, T1-weighted contrast enhanced MRI

Field strength: 14.1 tesla

Sequence & imaging parameters: First, T2-weighted images from the entire brain were acquired for adequate positioning of the grid used for hyperpolarized 13C acquisitions (repetition time 1200 ms, echo time 20 ms, slice thickness 1.8 mm, 2 averages, matrix 256 × 256, field of view 30 × 30 mm²). Hyperpolarized [1-13C]pyruvate and [13C]urea (0.35 mL, 80 mM and 78mM, respectively) solution was injected i.v. over a period of 12 s through the tail vein catheter. From the beginning of the i.v. injection of hyperpolarized [1-13C]pyruvate and [13C]urea, 2D dynamic chemical shift imaging (CSI) 13C data from the brain were acquired (repetition time 60 ms, echo time 1.2 ms, spectral width 2500 Hz, 128 points, 4 s temporal resolution, flip angle 10°; field of view 24 × 24 mm²; slice thickness 5 mm).

Next, for T1-weighted MRI, the dual tune 1H-13C volume coil (ØI = 40 mm) was removed and replaced by a 1H volume

only coil (ØI = 40 mm). T1-weighted images were acquired (repetition time 120 ms, echo time 2 ms, slice thickness 0.8 mm, 10 averages, matrix 256 × 256, field of view 20 × 20mm²) prior and after gadolinium diethylenetriamine pentaacetate (DTPA) injection (0.1 mL, 1 mmol/kg, Magnevist, Bayer).

Area of acquisition: For T2 weighted MRI, whole brain scan was used
For hyperpolarized 13C MR spectroscopy, the slice was centered on the center of the brain (hippocampus)
For T1 weighted MRI, whole brain scan was used

Diffusion MRI ☐ Used ☒ Not used

Preprocessing

Preprocessing software: Hyperpolarized 13C MRS imaging data was analyzed using the in-house SIVIC software (http://sourceforge.net/apps/trac/sivic/) and custom-built programs written in MATLAB (MATLAB R2011b, The MathWorks Inc.). The k-space dimensions were zero-filled by a factor of two resulting in a 16×16 matrix. Spectra were summed over time and a lorentzian shape was used to fit the hyperpolarized [1-13C]pyruvate, [1-13C]lactate and [13C]urea peaks on the sum spectrum. Then, area under the curve (AUC) of hyperpolarized [1-13C]pyruvate, AUC of hyperpolarized [1-13C]lactate and AUC of hyperpolarized [13C]urea lorentzian fits were measured for each voxel. hyperpolarized [1-13C]lactate-to-pyruvate ratio was calculated as the ratio of the AUC. AUC of hyperpolarized [13C]urea signal was normalized to the AUC of hyperpolarized [13C]urea from surrounding tissue containing blood vessels to account for variations in polarization levels and transfer time. Next, average from voxels containing cortex, corpus callosum, external capsule and hippocampus was calculated, and the obtained mean values were used to evaluate statistical significance between experimental groups. In addition, the mean μ and standard deviation σ of the hyperpolarized 13C lactate / pyruvate ratio from all animals were computed for each individual voxel. Next, the hyperpolarized 13C lactate / pyruvate ratio values (xi) for each animal was normalized by converting it to a z-score using the following formula: $z = (x_i - \mu) / \sigma$. Color heatmaps of hyperpolarized [13C]urea, hyperpolarized 13C lactate / pyruvate ratio and mean z-score for each experimental groups were generated using a linear-based interpolation of the 13C 2D CSI data to the resolution of the anatomical images using custom-built programs written in MATLAB and SIVIC.

Normalization: NA

Normalization template: NA

Noise and artifact removal: NA

Volume censoring: NA

Statistical modeling & inference

Model type and settings: NA

Effect(s) tested: NA

Specify type of analysis: ☐ Whole brain ☐ ROI-based ☒ Both

Anatomical location(s): whole brain, cortex, corpus callosum, external capsule and hippocampus

Statistic type for inference (See Eklund et al. 2016): NA

Correction: NA

Models & analysis

n/a	Involved in the study
☒	☐ Functional and/or effective connectivity
☒	☐ Graph analysis
☒	☐ Multivariate modeling or predictive analysis
